# A contrast between DEMATEL-ANP and ANP methods for six sigma project selection: a case study in healthcare industry

**DOI:** 10.1186/1472-6947-15-S3-S3

**Published:** 2015-09-04

**Authors:** Miguel A Ortíz, Heriberto A Felizzola, Santiago Nieto Isaza

**Affiliations:** 1 Department of Industrial Engineering, Universidad de la Costa CUC, Barranquilla, Colombia; 2 Department of Industrial Engineering, Universidad de la Salle, Bogotá, Colombia; 3 Department of Industrial Engineering, Universidad del Norte, Puerto Colombia, Colombia

**Keywords:** Six-Sigma project selection, Analytic Network process (ANP), Decision making trial and Evaluation Laboratory (DEMATEL), Healthcare

## Abstract

**Background:**

The project selection process is a crucial step for healthcare organizations at the moment of implementing six sigma programs in both administrative and caring processes. However, six-sigma project selection is often defined as a decision making process with interaction and feedback between criteria; so that it is necessary to explore different methods to help healthcare companies to determine the Six-sigma projects that provide the maximum benefits. This paper describes the application of both ANP (Analytic Network process) and DEMATEL (Decision Making trial and evaluation laboratory)-ANP in a public medical centre to establish the most suitable six sigma project and finally, these methods were compared to evaluate their performance in the decision making process.

**Methods:**

ANP and DEMATEL-ANP were used to evaluate 6 six sigma project alternatives under an evaluation model composed by 3 strategies, 4 criteria and 15 sub-criteria. Judgement matrixes were completed by the six sigma team whose participants worked in different departments of the medical centre.

**Results:**

The improving of care opportunity in obstetric outpatients was elected as the most suitable six sigma project with a score of 0,117 as contribution to the organization goals. DEMATEL-ANP performed better at decision making process since it reduced the error probability due to interactions and feedback.

**Conclusions:**

ANP and DEMATEL-ANP effectively supported six sigma project selection processes, helping to create a complete framework that guarantees the prioritization of projects that provide maximum benefits to healthcare organizations. As DEMATEL- ANP performed better, it should be used by practitioners involved in decisions related to the implementation of six sigma programs in healthcare sector accompanied by the adequate identification of the evaluation criteria that support the decision making model. Thus, this comparative study contributes to choosing more effective approaches in this field. Suggestions of further work are also proposed so that these methods can be applied more adequate in six sigma project selection processes in healthcare.

## Background

The application of six sigma methodology in the optimization of healthcare processes is a relevant aspect for medical industry since health caring has become in a complex problem where several factors converge such as: high operating costs in medical centres, high costs in medical treatments and drugs, high labour costs, increase of medical services demand, the establishment of health as a fundamental right; among others [1-3]. Upon giving solutions to each of these problems through Six Sigma approach; it is required to make effective decisions that make possible to focus the human and financial resources appropriately on projects whose contribution lead to the improvement of organizations performance [4-6].

One of the key factors of Six Sigma methodology is the correct selection of projects [7-10]. This process consists about identifying, prioritizing and selecting the project(s) that provide(s) the highest impact on organization goals[11], and is done through the evaluation of a set of criteria such as: Impact on product or service quality, impact on customer satisfaction, impact on revenue growth, project costs, project duration, risks, information reliability; among others[4,12-14].

The six sigma project selection involves the evaluation of different criteria by a set of professionals, who have a wide knowledge of the organization goals and key processes, and they are commonly considered as leaders with high level of responsibility [15,16]. Therefore, it is important to use decision making methods or model that make possible to increase the success probability[15]. That is why methods like Analytic Hierarchy Process (AHP), Analytic Network Process (ANP), Goal Programming, Delphi, Decision Making Trial and Evaluation Laboratory (DEMATEL) and Fuzzy Logic have been widely used for this purpose.

The Analytic Hierarchy Process is a technique that allows to modelling decision making processes through problem decomposition under a hierarchical structure composed by goals, criteria, sub-criteria and alternatives, in which a set of participants evaluates each of these components by pairwise comparisons[17,18].

One of the weaknesses of AHP is in the fact that does not allow to evaluating interrelations and influences between the elements that compose the decision making process. Hence, Saaty developed a general structure called Analytic Network Process (ANP)[19,20]. This method is a generalization of AHP and is currently used in decision making processes in which it is known that decision alternatives and criteria may have very strong interrelations and influences generating a high impact on the decision [21-24].

Even though ANP permits to evaluate the influence and interdependence, in some cases, this not understandable by decision makers; hence that DEMATEL starts plying a relevant role since it permits to have a better comprehension of the influences by the analysis of elements in cause and effect relationships[25,26]. DEMATEL is based on graphs theory, reason by which decision makers can have a better understanding of casual relationships that are characterized by being complex and, in some cases, imperceptible [27,28].

As a complementary strategy and with the purpose of improving the comprehension of the current decision making problems, there is an inclination towards the combination of different methods[29]. Specifically, for six sigma project selection, different combined techniques have been used such as: Fuzzy-AHP [30,31], Fuzzy-ANP [32], ANP-DEMATEL [4,33] and Delphi fuzzy [15], whose primary aim is to reinforce the methods previously mentioned.

In particular, we focus on the application of DEMATEL-ANP to select the most suitable six sigma project for a specific medical centre. We described how DEMATEL-ANP method was adapted to improve is effectiveness for application in healthcare processes. Finally, a comparative study between DEMATEL-ANP and ANP methods for six sigma project selection in a medical centre. This is done with the purpose of demonstrating how a combined technique leads to better results in decision making process.

## Methods

### Ethical considerations

Before beginning this research, the project methodology was presented and discussed with the chief executive and the ethics committee of the medical centre in study. As this study was supported in an interview with staff from the medical centre and did not require patient involvement, no formal approval by the committee was necessary. Finally, the participants of the medical centre in head of the chief executive, gave informed consent to participate in this study.

### Network definition

The six-sigma team identified a total of 6 project alternatives thanks to the analysis of user satisfaction with respect to different services of the medical centre, evaluation of key performance indexes (KPI´s) and internal process analysis. In the other side, 3 strategies, 4 criteria and 15 sub-criteria were defined with basis on the needs of this particular organization and healthcare sector. To identify each element of the network, a three-phased systematic method was realized. First, customer requirements were analysed and identified through a six-sigma tool called as VOC (Voice of customer). Then, organizational policies and strategies were studied; and finally, a project portfolio was defined taking into account the projects that could best improve the customer satisfaction and achieve the organization goals. Specifically, the main problems were identified in the departments of: drug inventory management (Project C1), emergency department (Project C2), internal medicine department (Project C3), ginecobstetric outpatient service (Project C4) and systems department (Project C5 and C6).

By the other side, each strategy was determined considering the organizational policies and strategic goals. First, BUSINESS EXCELLENCE, defined as the systematic improvement of the business performance based on the principles of customer focus, stakeholders satisfaction and administrative process [34]. Second, REVENUE GROWTH, identified as the increase in the revenues received by the company because of its services and finally, HIGH PRODUCTIVENESS, which establishes the relation between the incomes generated by the service and the amount of resources invested in its provision.

To determine the criteria and sub-criteria, a literature review was done to identify the key aspects to evaluate the effectiveness of six-sigma project [1,3-8].

At the end, the key aspects are summarized in four categories (clusters): Benefits, Opportunities, Project costs and Project risks.

The six-sigma team was composed by the chief executive of the medical centre, 1 industrial engineer who is co-author of this paper (MAO), and participants from financial, quality and information system departments who have a wide experience in healthcare management. MAO acted as the director and based on his experience about ANP and DEMATEL-ANP, designed the network, which was verified with the rest of the team in order to check it was understandable and clear.

**Figure 1:**
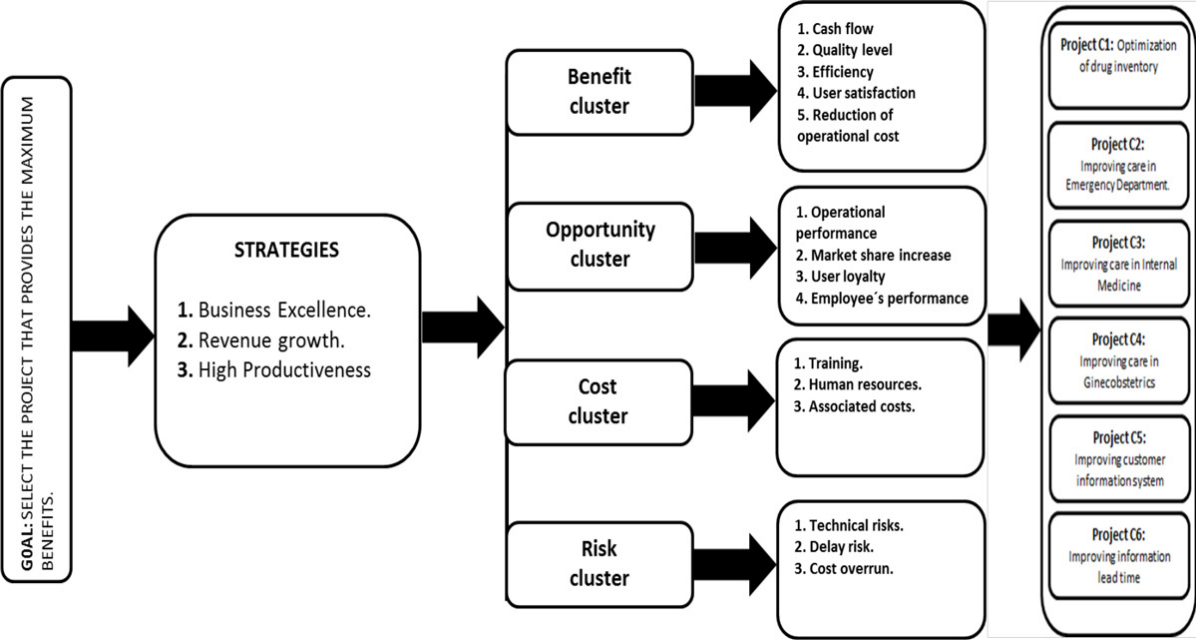
****Evaluation model for six sigma project selection****. Adapted from [4].

The 15 sub-criteria were organized into 4 criteria clusters and an evaluation model was designed (Figure 1) taking into account that a strategy cluster supported the GOAL achievement.

### Matrix design

For DEMATEL, a matrix was designed with the purpose of enabling six sigma team to make pairwise comparisons between each strategy and all of the other strategies, and between each sub-criterion and all of the other sub-criteria (within the same criterion). The information gathering tool designed for DEMATEL is shown in Table 1. In this case, strategy cluster was evaluated taking into account its elements (OE: Organizational Excellence, RG: Revenue Growing and HP: High Productiveness).

**Table 1:** Matrix design for DEMATEL.

	OE	RG	HP
**OE**		4	3

**RG**	3		3

**HP**	3	4	

For each pair of strategies o sub-criteria, participants were asked the following question: ¿How much influence does strategy/sub-criterion i have on strategy/sub-criterion j? Each participant responded by selecting one of the following judgments established for DEMATEL: no influence (0), low influence (1), medium influence (2), high influence (3) and very high influence (4).

As next step, the matrix for ANP was designed taking into account how a pair of elements contributes to its particular upper level criterion. The information gathering tool designed for ANP is shown in Table 2. In this case, cost cluster was evaluated taking into account its elements (AC: Associated costs, T: Training and HR: Human Resource).

**Table 2:** Matrix design for ANP.

Cost cluster	AC	T	HR
**AC**	1	9	8

**T**	1/9	1	1/9

**HR**	1/8	9	1

For each pair of strategies o sub-criteria, participants were asked the following question: ¿How important is strategy/sub-criterion i over strategy/sub-criterion j with respect to their particular upper level criterion k? Each participant answered according to Saaty´s 1-9 point [34] scale where 1 represents equal importance and 9 represents extreme importance of one strategy/sub-criterion over another. On the other hand, respondents were also asked the question: ¿How important is project alternative i over project alternative j with respect to a strategy or sub-criterion l? Each participant responded according to the same Saaty´s 1-9 scale previously explained.

Then, DEMATEL and ANP matrixes were completed until finishing the entire evaluation model.

### Decision Making Trial and Evaluation Laboratory (DEMATEL)

After having made all the comparisons, matrixes have to be normalized. It is worth noting that these comparisons form *n × n *matrixes A whose elements are denoted as *a*_*ij*_. Then, the normalized direct-relation matrix is called M [35-37] and is obtained from the product of the original matrix with a constant *k *which is the minimum value between the sums across columns and rows of the inverse original values of matrix (A) respectively (See formulas (1) and (2)):

(1) M=k⋅A

(2) $$k=\text{min}\left(\frac{1}{\underset{1\leq i\leq n}{\text{max}}\overset{n}{\underset{j=1}{\sum }}|a_{ij}|},\frac{1}{\underset{1\leq j\leq n}{\text{max}}\overset{n}{\underset{i=1}{\sum }}|a_{ij}|}\right)i,j\in \left\{1,2,3...,n\right\}$$

As next step, the total-relation matrix **(S) **total relation matrix is calculated with basis on matrix M and the identity matrix I (See formula (3)). This matrix computes the overall influence from one factor to the others and viceverse, this is the base to define the overall degree of influence of each factor and hence, to define and prioritize factors (criteria).

(3) $$S=M+M^{2}+M^{3}+\ldots =\overset{\infty }{\underset{i=1}{\sum }}M^{i}=M{\left(I-M\right)}^{-1}$$

Then, factors are classified in dispatchers and receivers. Dispatchers are considered to be more influent in the decision and hence, are prioritized, Receivers on the other side, are assumed to be of less priority [6,27]. With the values of D - R where R is the column sum and D is the row sum of matrix S (See Formulas (4) - (6)), the interrelations can be estimated. If a strategy or sub-criterion has a positive value of D - R, then the strategy or sub-criterion has a high influence on another strategy or sub-criterion respectively; therefore, it is assumed as a high-priority sub-criterion/strategy called "dispatcher". By the other side, if a strategy or sub-criterion has a negative value of D - R, then the strategy or sub-criterion receive influence from another strategy or sub-criterion severally; hence, it is categorized as a low-priority sub-criterion/strategy called "receiver".

(4) $$S={\left[s_{ij}\right]}_{nxn},i,j\in \left\{1,2,3,\ldots ,n\right\}$$

(5) $$D=\overset{n}{\underset{j=1}{\sum }}s_{ij}$$

(6) $$R=\overset{n}{\underset{i=1}{\sum }}s_{ij}$$

The values of D + R have also a meaning. This value indicates the relation degree between each sub-criterion/strategy with others. The sub-criterion/strategy with the highest D + R value is more related to the others; while little values of D + R show a weak relation with the rest. As final step, the inner dependence matrix is calculated, for which the sum of each column in total-relation matrix is equal to 1 by normalization.

### Analytic Network Process (ANP)

According to Saaty theory, the resulting matrixes in ANP have a series of properties [34]:

1. The component (a_ij_) related to the ratio between the relative importance of the sub-criterion or strategy "i" (N_i_) and sub-criterion or strategy "j" (N_j_).

2. The component a_ji _is the reciprocal of a_ij _adopting the reciprocity of judgment (whether N_i _is 5 times more important than N_j_, then N_j _should be 1/5 of N_j_).

3. The component a_ii _is equal to 1.

4. The matrix A is adopted as a transitive matrix, which means that "$\forall i,j,k\in \left(1;n\right),a_{ij}=a_{ik}*a_{kj}$ "by definition of aij (Formula (7)).

(7) $$a_{ij}=\frac{N_{i}}{N_{j}}=\frac{N_{i}}{N_{k}}*\frac{N_{k}}{N_{j}}=a_{ik}*a_{kj}$$

This property indicates that if a sub-criterion or strategy "i" is considered as twice as important as sub-criterion or strategy "j" (N_i _= a_ij _* N_j_), and sub-criterion or strategy "j" is four times more important than sub-criterion or strategy "k" (N_j _= a_jk _* N_k_), then sub-criterion "i" should be qualified eight times (two times four) more important than sub-criterion or strategy "k" (N_i _= a_ik _* N_k_, with a_ik _= a_ij _* a_jk_).

As next step, the final decision supermatrix is created. This supermatrix provides the absolute weight of each alternative for solving the problem. In this stage of the ANP methodology, a portioned matrix is built from the pairwise comparisons previously made. First, original values obtained in Saaty's scale must be transformed in a weighted supermatrix, this is, all columns must sum to unity. Then a *Limit Supermatrix *is constructed in order to guarantee that the weights are stable, this procedure implies that the weights are raised to limiting powers until a convergence is observed [38].

Once the convergence of the supermatrix in ensured the stabilized weights are normalized by blocks [39], this procedure is done for all the blocks defined in the decision network. The absolute importance (weight) is determined by the total weight of the column representing each alternative of solution for the problem [40,41], according to this, the best alternative is the one with the largest weight (See formula (8)).

(8) $$\frac{'}{\begin{array}{c} \;\;S_{1}\\ W=S_{2}\\ \;\;\vdots \\ \;\;S_{N} \end{array}}\frac{\begin{array}{cc} S_{1}\; & \begin{array}{ccc} S_{2}\; & \;\ldots & \;S_{N} \end{array} \end{array}}{\left[\begin{array}{cccc} W_{11} & W_{12} & \ldots & W_{1N}\\ W_{12} & W_{22} & \ldots & W_{2N}\\ \vdots & \vdots & \ldots & \vdots \\ W_{1N} & W_{2N} & \ldots & W_{NN} \end{array}\right]}$$

### Relative importance of sub-criteria within each criterion and strategies within strategy cluster

Saaty proved [34] that when the matrix A satisfies the properties previously indicated for ANP judgement matrixes, only one real eigenvalue (λ) exists. In this way, the eigenvector associated with this eigenvalue, represents the relative weight of each sub-criterion or strategy to each of the other sub-criteria or strategies respectively. This relative weight of a sub-criterion or strategy "i" within the criteria or strategy cluster m is called local weight $LW_{i}^{m}$. By the other side, if the comparisons of a matrix are not completely consistent, the matrix has more eigenvectors and none of them is proportional to all the columns. Consequently, the eigenvector with the highest eigenvalue (λ_máx_) is selected and the normalized elements represent the relative weight of each strategy or sub-criterion.

### Consistency calculation

Inconsistency is the result of the loss of interest or distractions. If this happens, the comparisons have to be made again. This inconsistency affects the reliability of the decision; however, some inconsistency is expected. In this case, the participants' consistence was calculated through the consistency index (CI) [42]. This index is equal to zero when the judgements are fully consistent (λ_máx_= n). According to literature, the CI is divided by random index (RI) whose values for 2 ≤ n ≤ 10 are shown in table 3[42]. This ratio is called consistency ratio (CR). A CR value ≤ 0.1 is considered appropriate [43] - [44].

**Table 3:** Values of Random Index (RI).

n	2	3	4	5	6	7	8	9	10
**RI**	0	0,58	0,9	1,12	1,24	1,32	1,41	1,45	1,51

### Category importance per participant

By using the same algorithm to the criteria, it was possible to determine their relative weight. The relative importance of a criterion *m *will be recalled as relative weight RW^m^. These weights reflect the consensus developed by the participants of the six-sigma team in the purpose of achieving a group decision. Each category was evaluated with respect to the model GOAL. In ANP, the category importance describes how relevant each criterion is with respect to the other at the moment of selecting the six-sigma project that provides the best benefits. In DEMATEL, it refers to how a particular criterion influences on other at the time of making the decision. Finally, RW^m ^values are used to calculate the global importance of each strategy and sub-criterion that makes part of the six-sigma project selection model as described in next section.

### Global importance of each strategy and sub-criterion per participant

The relative weight of a strategy or sub-criterion i compared to the rest of the strategies and sub-criteria respectively (not only in the same cluster), is identified as global weight of the strategy or sub-criterion i (GW_i_). GWs are estimated by multiplying the local weight of the strategy or sub-criterion by the weight of the root component into the level of the network (See formula (9)).

(9) $$GW_{i}=LW_{i}^{k}*RW^{k}$$

### Comparison between ANP and DEMATEL-ANP

Comparison of both methods is based on a set of required characteristics of the used techniques for six sigma project selection process in healthcare. The factors that were considered in this comparative study were: adequacy to changes of six sigma project alternatives, agility in the decision process and adequacy to changes of strategies and sub-criteria.

*Adequacy to changes of six sigma project alternatives *refers to the possible inclusion or exclusion of six sigma project alternatives in the evaluation process without causing inconsistencies in the project ranking. By the other side, *agility in the decision process *represents the required amount of judgements of decision makers at the moment of collecting data. Taking into account the Multicriteria Decision Making (MCDM) technique, the number of sub-criteria and six-sigma project alternatives, decision making process could be very time-consuming. Finally, *adequacy to changes of strategies and sub-criteria *denotes the inclusion or exclusion of sub-criteria due to the presence of new healthcare regulations issued by government or other interests from the rest of the stakeholders. In this case, the MCDM technique should be robust enough not to generate inconsistencies in the sub-criteria ranking.

### Feedback of six-sigma team

Finally and with the purpose of comprehending the reasons behind the prioritization of strategies, sub-criteria and six sigma project alternatives, the results were discussed with the participants of the Six Sigma Team and the chief executive of the medical centre. Each participant felt good and comfortable at the moment of making the comparisons. In addition, the participants expressed the methods were completely understandable and they did not generate any confusion.

### Six-sigma team

Six professionals from Quality Management Department (2), Financial Department (2), General Management (1) and User Service Department (1), each one of them with more than 15 years of experience, working in the same medical centre, were the participants of six sigma team and who filled up the judgment matrixes previously shown. None of these professionals is one of the authors of this paper. All these professionals had a wide knowledge of all this medical centre departments; however, each was asked to answer with respect to the department in which they were working in order to guarantee a global perception of the medical centre at the moment of evaluating the six sigma project alternatives.

## Results

Table 4 shows the dispatchers and receivers per each cluster. The global and local weights of each strategy and sub-criterion in combined technique DEMATEL- ANP are reported in Table 5. Table 6 shows the global weights of decision alternatives. Table 7 show the consistency ratios for DEMATEL-ANP matrixes respectively. All matrixes achieved the required threshold (CR≤ 0.1)

**Table 4:** D + R and D - R values of each strategy and sub-criterion.

Cluster	D + R	D - R	Dispatcher	Receiver
**Strategies**				
Organizational excellence	13,222	0,842	X	
Revenue growth	12,938	-1,424		X
High productiveness	12,926	0,582	X	
**Benefit**				
Cash flow	12,045	-0,861		X
Quality level	12,397	-0,375		X
Efficiency	13,764	0,728	X	
User satisfaction	13,931	0,165	X	
Reduction of operational cost	12,063	0,343	X	
**Opportunity**				
Operational performance	10,494	0,556	X	
Market share increase	4,852	-2,407		X
User loyalty	9,75	0,206	X	
Employees´ performance	9,639	1,645	X	
**Cost**				
Training	4,756	0,89	X	
Human resources	2,808	0		
Associated costs	4,754	-0,89		X
**Risk**				
Technical risks	7,297	-0,46		X
Delay risk	7,163	-0,618		X
Cost overrun	15,39	1,078	X	

**Table 5:** Local and global weights of evaluation sub-criteria in DEMATEL-ANP method (CR ≤ 0.1).

Cluster	GW	LW
**Benefit (RW = 0,25)**		
Cash flow (S_1_)	0,02414	0,09655
Quality level (S_2_)	0,02812	0,11248
Efficiency (S_3_)	0,02741	0,10965
User satisfaction (S_4_)	0,02345	0,09382
Reduction of operational cost(S_5_)	0,02187	0,0875
**Opportunity (RW = 0,25)**		
Operational performance (S_6_)	0,0356	0,14241
Market share increase (S_7_)	0,0212	0,08482
User loyalty (S_8_)	0,0321	0,12841
Employees´ performance (S_9_)	0,03609	0,14436
**Cost (RW = 0,25)**		
Training (S_10_)	0,0289	0,11562
Human resources(S_11_)	0,05471	0,21883
Associated costs(S_12_)	0,04139	0,16555
**Risk (RW = 0,25)**		
Technical risks(S_13_)	0,04029	0,16116
Delay risk(S_14_)	0,0396	0,1584
Cost overrun(S_15_)	0,04511	0,18044

**Table 6:** Global weights of decision alternatives in DEMATEL-ANP method (CR ≤ 0.1).

Decision alternative	GW
**Project C1: **Optimization of drug inventory	0,05339
**Project C2: **Improving care in Emergency Department	0,0892
**Project C3: **Improving care in Internal Medicine	0,09166
**Project C4: **Improving care in Ginecobstetrics	0,1168
**Project C5: **Improving customer information system	0,0853
**Project C6: **Improving information lead time	0,06366

**Table 7:** Consistency ratio (CR) per cluster.

Cluster	CR
Strategy	0,01
Benefit	0,03
Opportunity	0,09
Cost	0,04
Risk	0,07

**Figure 2:**
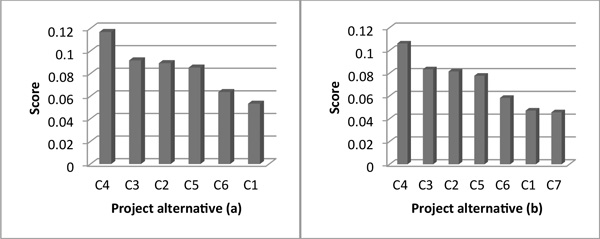
**Test results for change of alternatives - DEMATEL-ANP**.

**Figure 3:**
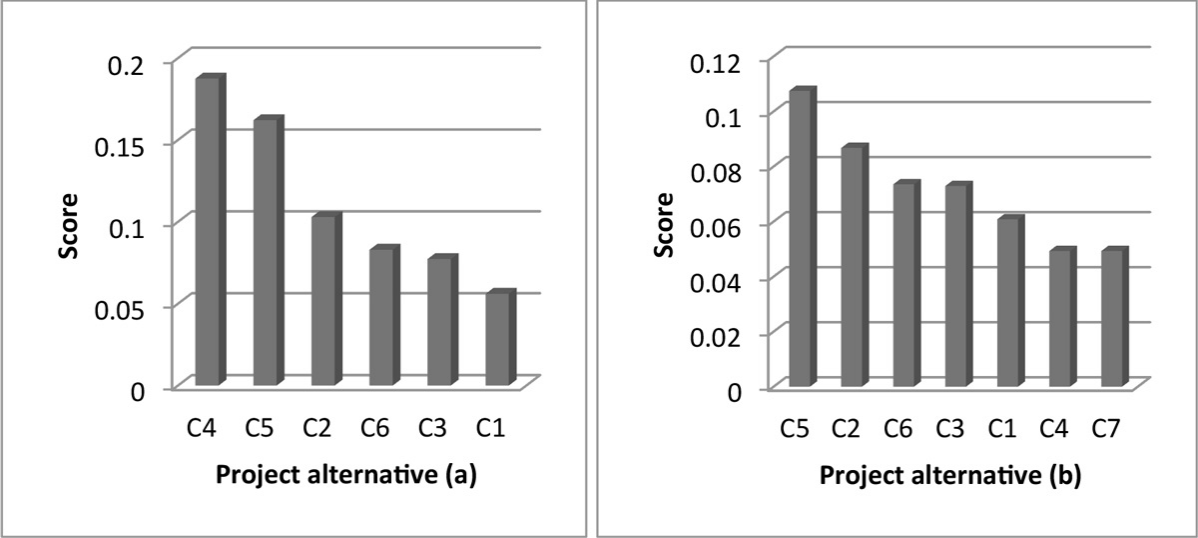
**Test results for change of alternatives - ANP**.

**Figure 4:**
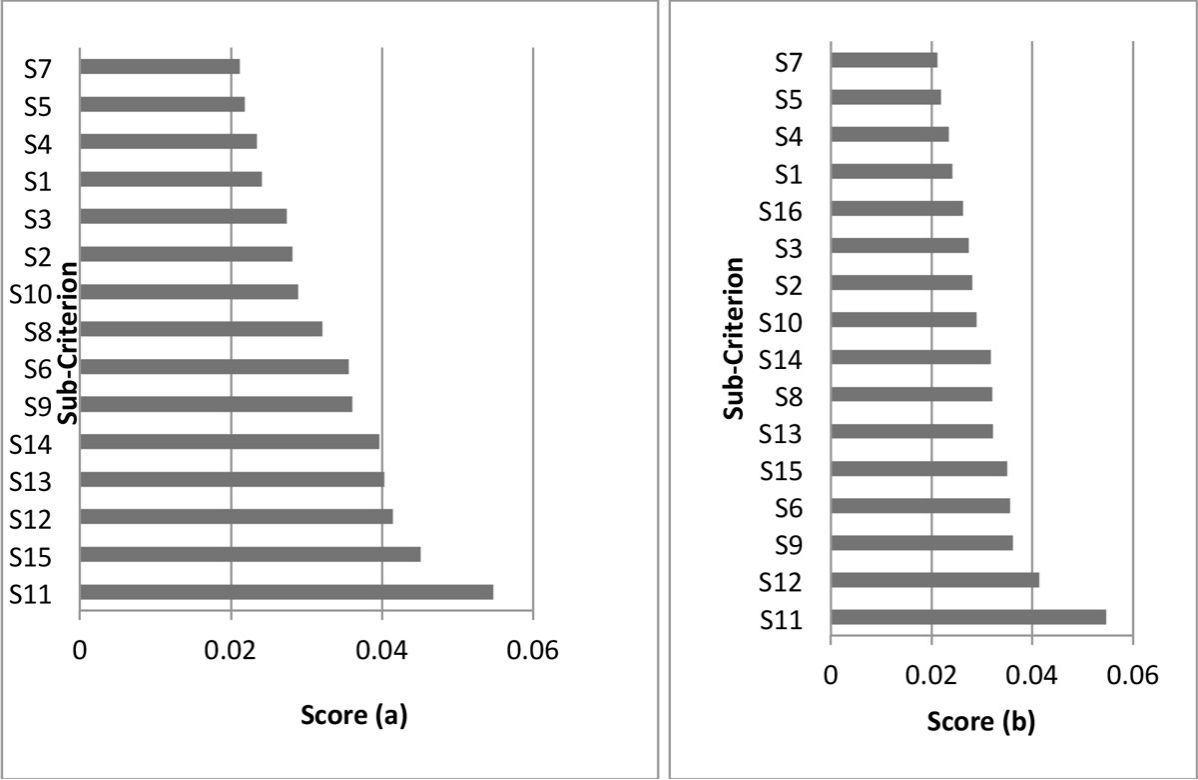
**Test results for change of sub-criteria - DEMATEL-ANP**.

**Figure 5:**
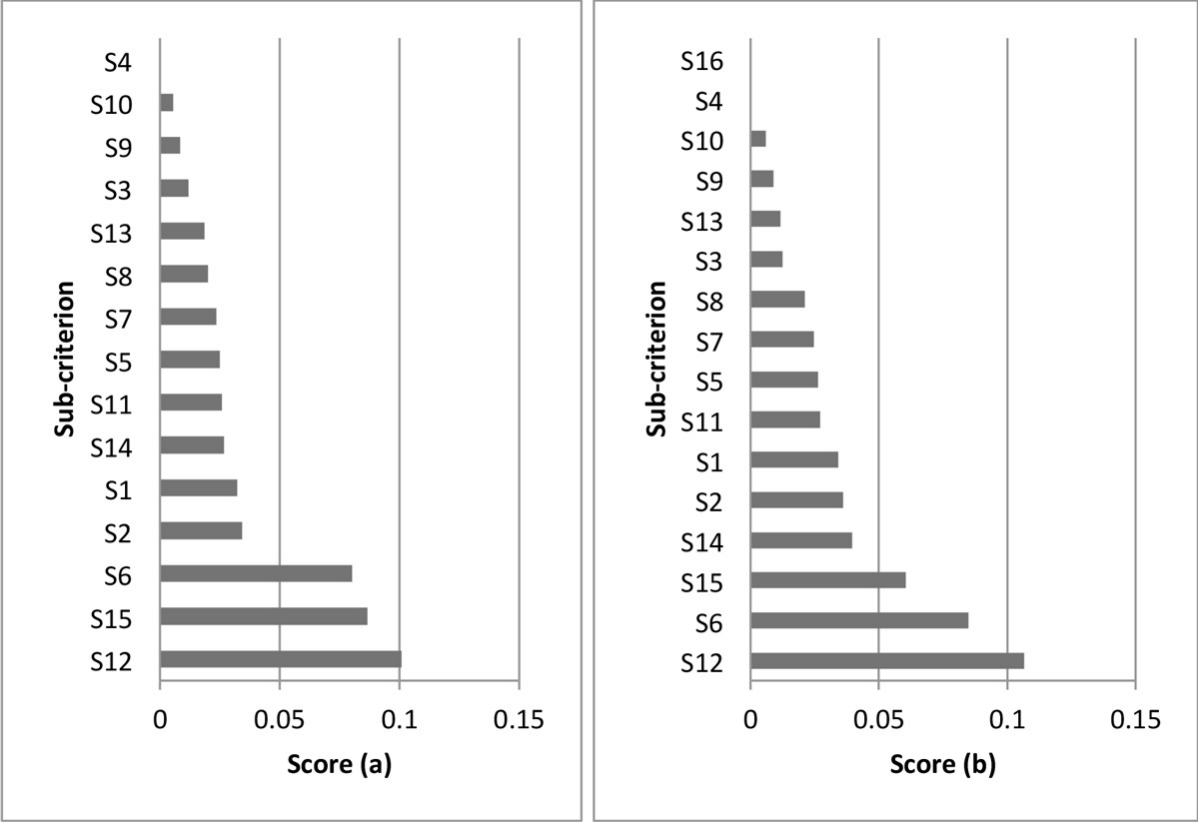
**Test results for change of sub-criteria - ANP**.

Figure 2 and 3 describe the test results of adequacy to changes of alternatives in both methods. Figure 4 and 5 show the results of adequacy to changes of criteria in both methods. Then, the agility in the decision process is analysed for both methods. Finally, in Table 8 a summarized comparative analysis of ANP and DEMATEL-ANP.

**Table 8:** Summarized comparative analysis between DEMATEL-ANP and ANP.

Comparison parameter	Comparison between DEMATEL-ANP and ANP
Adequacy to changes of alternatives	DEMATEL-ANP is consistent in the project alternative ranking while ANP does not generate consistent preference order.
Adequacy to changes of sub-criteria	When new sub-criterion is included, DEMATEL-ANP is more sensible than ANP since it takes into account the interrelations and feedbacks.
Agility in the decision process	When a project alternative is included, the number of additional judgements is the same in both methods while if a sub-criteria is included, ANP will require less comparisons than DEMATEL-ANP

## Discussion

In this paper, we presented the results of a study on the application of ANP and DEMATEL-ANP besides to a comparative analysis between them so that, researchers and practitioners can choose more accurate approaches for six sigma project selection process in healthcare sector. As a case study, we focused on strategies, criteria and sub-criteria related to the selection of six sigma projects in a medical centre. At the moment of applying DEMATEL, D + R and D - R values are determined as shown in Table 4. This is done with the purpose of identifying the interrelations between the elements of each decision cluster at the time of decision making. It is seen that ORGANIZATIONAL EXCELLENCE and HIGH PRODUCTIVENESS have a high influence on REVENUE GROWTH in strategy cluster. By the other side, in benefit cluster, EFFICIENCY, USER SATISFACTION and REDUCTION OF OPERATIONAL COST sub-criteria have a high impact on CASH FLOW and QUALITY LEVEL. As for opportunity cluster, it is observed that MARKET SHARE INCREASE receives influence by the rest of the sub-criteria. On the other hand, TRAINING influences on ASSOCIATED COSTS criterion in cost cluster. Finally, TECHNICAL RISKS and DELAY RISKS are affected by COST OVERRUN in risk cluster.

Regarding local weights within the criterion of *benefit *(Table 5), quality level was considered the most important sub-criterion. This reflects the fact that healthcare sector is mainly worried about caring patients with high standards of quality. By the other side, reduction of operational cost was considered the least important by the six sigma team. At the moment of discussing these results with the medical centre director and the six sigma team, it emerged that the local government had determined a framework to measure the quality of the services offered by all medical centres, reason by which all these kinds of organizations wanted to improve their quality management systems.

Regarding local weights within the criterion of *opportunity *(Table 5), employees' performance was ranked as the most important sub-criterion. This shows the need of increasing the skills of the medical centre workforce since it has a greater intervention on the healthcare services with respect to the other resources. It is worth noting that workforce has a big influence on the quality level of the caring processes and determine the perception of the users. On the other hand, market share increase was considered as the least important sub-criterion by the participants.

With respect to the local weights in the criterion of *cost *(Table 5), "human resources" was considered as the most relevant sub-criterion. This is related to the fact that six sigma projects are composed by a high cost of labour since it requires highly qualified professionals in statistics and quality management, besides the healthcare experts who are essential at the moment of analysing and designing improving strategies for the process in study. By the other side, cost of training was ranked as the least relevant criterion by the responders.

Regarding local weights within the criterion of *risk *(Table 5), cost overrun was chose as the most significant sub-criterion. This result can be linked with the fact in which there is an economic crisis in healthcare sector. All the medical centres are very careful when investing on projects with a high risk level of budget overrun since these projects could affect the financial sustainability of these organisations. Meanwhile, delay risk was identified as the least relevant sub-criterion in this category.

With regard to global weights, Table 5 shows that the top five important sub-criteria for selecting the most suitable six sigma project are: *human resources, cost overrun, associated costs, technical risks *and *delay risk*. It is seen that the six sigma team did not consider any opportunity or benefit sub-criterion as relevant for this decision. The most important criteria were selected from cost and risk cluster. It is noticed that all the criteria that are within risk cluster are included in the top five. This fact could be related to some investment policies that healthcare companies have assumed to face economic restrictions; therefore they aim to get the best results at minimum risks and costs.

In regard to global weights of decision alternatives and their contribution to the goal, *improving care in Ginecobstetrics *obtained the highest score. This is explained since the main market of this medical centre is composed by pregnant women who ask for different services that are linked to their status. Consequently, most of its incomes come from these kinds of services; hence, if a positive impact is generated in *Ginecobstetrics*, it resulted in a positive impact in the entire medical centre.

As next step, a comparative analysis of DEMATEL-ANP and ANP is done in the context of six sigma project selection in healthcare. The following factors were considered at the time of doing the comparison: adequacy to changes of alternatives, adequacy to changes of sub-criteria and agility in the decision process [41].

### Adequacy to changes of alternatives

In the six sigma project selection for healthcare companies, the evaluation of a different set of six sigma projects might require the inclusion or exclusion of alternatives. In this case, the selection tool must generate a consistent ranking of alternatives.

In the DEMATEL-ANP application case, with 3 strategies, 4 criteria, 15 sub-criteria and their respective importance, the ranking was C_4 _> C_3 _> C_2 _> C_5 _> C_6 _> C_1_, as illustrated in Figure 2a. To test the DEMATEL-ANP method, an additional project alternative (C_7_) was evaluated. Complementary tests were performed, each one with an additional alternative with a rating equal to one of the six initial decision alternatives.

In DEMATEL-ANP, the results have shown no relevant changes in the project alternative ranking. However, there are some variations in the alternative final scores [46-48].This can be explained in the fact that the inclusion of a new project alternative brings about new considerations by the six-sigma team; although did not bring about a change in the preference order as seen in Figure 2b.

On the other hand, in the ANP application case, there were considerable modifications in the order of preferences. It is observed that when the additional six sigma project alternative has a rating equal to the best alternative (C4 in Figure 3a); the resulting project ranking varies significantly. In this case, what was the best alternative, C4, becomes the penultimate option, as shown in Figure 3b; which is not expected in six sigma project selection problems.

### Adequacy to changes of criteria

If there are some changes in healthcare sector, we might also need to change some of the initial criteria used to evaluate six sigma projects. For this case, the criteria importance ranking generated by the selection method must be consistent.

In the DEMATEL-ANP application case, with 15 sub-criteria and respective contribution, the sub-criteria importance ranking was S_11 _> S_15 _> S_12 _> S_13 _> S_14 _> S_9 _> S_6 _> S_8 _> S_2 _> S_3 _> S_1 _> S_4 _> S_5_. To verify the effect of adding a new sub-criterion, complementary tests were carried out, each one with the inclusion of a new sub-criterion that assumes a weight equal to one of the fifteen initial sub-criteria.

In DEMATEL-ANP and through the application of the tests that include a new sub-criterion, it is observed there are relevant changes in the preference order of the sub-criteria. In this case, what was the second project alternative in the ranking (S_15 _in Figure 4a), now becomes the fifth option, as shown in Figure 4b. These kinds of modifications can be linked to the regulations or sector goals. These events motivate healthcare organizations to prioritize certain factor over the rest. By the other side, in the ANP application case, it is seen the same performance that happened in DEMATEL-ANP at a lower degree. For instance, what was the second project option in the preference order (S_15 _in Figure 5a), becomes the third option, as shown in Figure 5b. This is due to the fact of ANP does not take into account the existing interrelations between sub-criteria which helps to reduce the impact on the consistency of the selection method.

### Agility in the decision process

This factor considers the number of judgments required from the six sigma team in both methods. In the application case, the DEMATEL-ANP method required 550 judgments while ANP required 500 under a structure composed by: 3 strategies, 15 sub-criteria and 6 project alternatives. If an additional project alternative is included, the number of pairwise comparisons per each method increases in 12 judgments for each sub-criterion. However, if a sub-criterion is included, DEMATEL-ANP method will require 30 additional besides the judgements that are generated depending on the cluster size in which the sub-criterion is included. For example, if the additional sub-criterion is included in cost cluster, 6 more judgements will be asked for a total of 36.

By the other side, ANP will just require 30 additional pairwise comparisons. Nevertheless, this fact represents more slowness in decision making process, reason by which some strategies should be designed in order to avoid loss of interest or distraction at the moment of implementing them.

The comparative analysis of DEMATEL-ANP and ANP has shown some relevant outcomes that should be taken into account to align the method to the particular characteristics of the six sigma project selection. The results expose the analysis of three factors that are valid for the context of this selection process. For other decision making problems, changes of alternatives, changes of sub-criteria and agility could be also important; so that the conclusions that are generated from these analysis.

Table 8 presents a summary of the findings. With regard to *adequacy to changes of alternatives and sub-criteria*, it can be observed that DEMATEL-ANP has a better performance since it takes into account the feedback and interrelations between sub-criteria. This fact results in a consistent project ranking and sensitivity to the different changes of sub-criteria assumed, in this case, by the medical centre and healthcare sector. On the other hand, ANP is better than DEMATEL-ANP in regard to the *agility in the decision making process*; however, DEMATEL-ANP can be combined with approaches related to the fact of reducing slowness, lack of interest and distraction; reason by which this disadvantage becomes irrelevant.

## Conclusions

This paper presented a new study comparing DEMATEL-ANP and ANP methods in regard to three factors that are particularly important to the problem of six sigma selection in healthcare industry. The primary aim of this research consisted about verifying the contribution of the combined technique DEMATEL-ANP on the decision making process with the purpose of getting better results. The performance of the methods concerning changes of alternatives or sub-criteria and agility in decision making process was evaluated through different tests based on inclusion of alternatives or sub-criteria. With regard to agility in decision making process, several tests were applied considering different scenarios of sub-criteria and decision alternatives. In this sense, the use of this hybrid method presented a better performance in comparison with ANP technique at the time of evaluating influences and generating consistent results. This demonstrates that it is necessary to use combined techniques, even more, when these techniques are specialized in a specific aspect like DEMATEL at the moment of verifying interrelations and feedback.

By the other side, one of the most practical aspects to highlight is in the fact that the selected Six Sigma project exceeded the expectations in regard to the results and impact on the medical centre where it was implemented. This is mostly due to the decision making process, from the definition of project alternatives, strategies, criteria and sub-criteria to the evaluation process. This process was developed in a clear and systematic way, highly supported in the methodology structure that is based on the combined technique DEMATEL-ANP. This should be considered before investing on six sigma projects even more there are strong economical restrictions in healthcare industry.

For future work, additional alternatives should be explored such as: FUZZY DEMATEL-ANP or FUZZY AHP-DEMATEL applied on the selection of six sigma projects in healthcare sector. This is done with the purpose of evaluating how much fuzzy approach can contribute to the management of linguistic variables in the decision making process.

## Abbreviations

DEMATEL: Decision Making Trial and Evaluation Laboratory.

ANP: Analytic Network Process.

AHP: Analytic Hierarchy Process.

VOC: Voice of customer.

OE: Organisational Excellence.

RG: Revenue Growing.

HG: High Productiveness.

AC: Associated costs.

T: Training.

HR: Human Resource.

CI: Consistency Index.

RI: Random Index.

CR: Consistency Ratio.

MCDM: Multicriteria Decision Making.

## Competing interests

The authors declare that they have no competing interests.

## Authors' contributions

MAO, HAF and SNI conceived this research. MAO and HAF designed the evaluation model for six sigma project selection and the matrixes. MAO, HAF and SNI analysed the data and presented the results. MAO led the evaluation process, prepared the ethical application and presented the results to the medical centre director and the participants. MAO, HAF and SNI discussed the results with basis on previous contributions related to this study. All the authors contributed to the paper. All the authors read and approved the final document.

## Declarations

Publication charges for this article have been funded by Universidad de la Costa CUC, Universidad de la Salle and Universidad del Norte.

This article has been published as part of *BMC Medical Informatics and Decision Making *Volume 15 Supplement 3, 2015: Multidimensional, multidiscipline and shared decision making in healthcare and eHealth. The full contents of the supplement are available online at http://www.biomedcentral.com/bmcmedinformdecismak/supplements/15/S3.
